# High-resolution infrared thermography: a new tool to assess tungiasis-associated inflammation of the skin

**DOI:** 10.1186/s41182-017-0062-9

**Published:** 2017-09-15

**Authors:** Angela Schuster, Marlene Thielecke, Vaomalala Raharimanga, Charles Emile Ramarokoto, Christophe Rogier, Ingela Krantz, Hermann Feldmeier

**Affiliations:** 1 Institute for Microbiology and Hygiene, Charité University Medicine, Berlin, Germany; 2 Institute Pasteur de Madagascar, Antananarivo, Madagascar; 3 Clinical Sciences Department, Institute for Tropical Medicine, Antwerp, Belgium; 4 Skaraborg Institute of Research and Development, Skövde, Sweden

## Abstract

**Background:**

Tungiasis is highly prevalent in low- and middle-income countries but remains often under diagnosed and untreated eventually leading to chronic sequels. The objective of the study was to assess whether tungiasis-associated inflammation can be detected and quantified by high-resolution infrared thermography (HRIT) and whether after removal of the parasite inflammation resolves rapidly.

**Methods:**

Patients with tungiasis were identified through active case finding. Clinical examination, staging, and thermal imaging as well as conventional photography were performed. In exemplary cases, the embedded sandfly was extracted and regression of inflammation was assessed by thermal imaging 4 days after extraction.

**Results:**

The median perilesional temperature was significantly higher than the median temperature of the affected foot (rho = 0.480, *p* = 0.003). Median perilesional temperature measured by high-resolution infrared thermography was positively associated with the degree of pain (rho = 0.395, *p* < 0.017) and semi-quantitative scores for acute (rho = 0.380, *p* < 0.022) and chronic (rho = 0.337, *p* < 0.044) clinical pathology. Four days after surgical extraction, inflammation and hyperthermia of the affected area regressed significantly (rho = 0.457, *p* = 0.005). In single cases, when clinical examination was difficult, lesions were identified through HRIT.

**Conclusion:**

We proved that HRIT is a useful tool to assess tungiasis-associated morbidity as well as regression of clinical pathology after treatment. Additionally, HRIT might help to diagnose hidden and atypical manifestations of tungiasis. Our findings, although still preliminary, suggest that HRIT could be used for a range of infectious skin diseases prevalent in the tropics.

**Trial registration:**

ISRCTN11415557, Registration date: 13 July 2011.

## Background

Tungiasis is a parasitic skin disease caused by the penetration of female sand fleas (*Tunga penetrans*) into the human epidermis. The burrowed flea increases its volume by a factor of 2000 within 2 weeks after penetration, eventually reaching a diameter of up to 10 mm [1]. Burrowed sand fleas exercise pressure on the surrounding tissue triggering an acute inflammatory response characterized by intense erythema, oedema, pain and pruritus [2, 3]. Usually, adults try to extract penetrated sand fleas by means of needles, pins or thorns, which result, almost constantly, in bacterial superinfections [4], caused by aerobic and anaerobic bacteria, including Clostridia [5]. These infections have the potential to cause severe complications such as cellulitis and tetanus [6] or could lead to mobility restrictions [7]. Tungiasis belongs to the neglected tropical diseases (NTDs), and it is endemic in many countries in Central and South America, in the Caribbean and in Sub-Saharan Africa [8–11].

Classical presentations of penetrated sand fleas are easily diagnosed, but hyperkeratinisation and other changes of the skin frequently occurring in patients who experience repeated infection may hamper the diagnosis, particularly when the skin is heavily pigmented.

High-resolution infrared thermography (HRIT) is a digital imaging technique in which a camera detects radiation of the electromagnetic spectrum within wavelengths from 900 to 14,000 nm. The emitted thermal radiation of a surface is measured and translated into a matrix of temperature measurements of this surface. Hence, HRIT can provide a visual map of skin temperatures in real time. Since inflammation of the skin or deeper tissue layers cause an increase in skin temperature, HRIT can allow the immediate assessment of inflammation of the skin [3, 6, 12].

The aims of this exploratory study were to map and quantify the spread of tungiasis-associated inflammation and to evaluate the resolution of inflammation after removal of the parasite.

## Methods

### Study area and study population

The study was performed in Tanambe II and Tanambaovao, two resource-poor rural communities, located in Andasibe county, Moramanga district, 160 km east of Antananarivo, the capital of Madagascar.

The inhabitants of the two villages are subsistence farmers and cultivate rice, cassava and fruits on small fields adjacent to the villages. Some villagers earn an extra wage as tourist guides in the nearby national park. Most houses are constructed from wood; some are placed on stilts. Chicken, pigs, dogs and cats are kept in the compound. Closed shoes are only worn irregularly and considered uncomfortable compared to walking barefoot or in flip flops. Besides, many families cannot afford shoes for all household members. A detailed description of the study area and population has been published elsewhere [13].

Tungiasis is highly prevalent in the region. At the time of this study, the prevalence of tungiasis in the study area in the general population was 72.9%, 95%CI (68.6–77.2) (M. Thielecke, unpublished observation, 2011).

### Study design and protocol

This qualitative, descriptive study was part of a comprehensive project on the epidemiology and prevention of tungiasis at the community level. For the HRIT substudy, patients were identified with active case finding by MT, AS and VR. Clinical examination and thermal imaging were carried out by MT and AS at the patient home in a shadowy place on the compound. Only when patients preferred privacy, thermal imaging was performed inside the house. Clinical examination and HRIT were limited to the feet. A total of 36 consecutive patients were identified and included in the study. To keep the workload manageable, a maximum of eight patients a day was recruited. The number of patients was limited by the availability of the HRIT camera.

Embedded sand fleas were removed surgically under sterile conditions by an experienced local health professional after baseline examination in nine patients. The wounds were appropriately dressed, and the patients were advised to wear closed shoes. HRIT was repeated immediately after the removal of the parasite as well as 4 days later. In all the other 27 patients, the embedded sand fleas were removed in the same manner at the end of the recruitment period.

Both feet were carefully examined with the aid of a dermatoscope allowing a tenfold magnification (Heine, Delta 20 T, Herrsching, Germany). Lesions were counted and classified according to the *Fortaleza Classification* described in Table 1. Lesions in stadiums I–IIIb where considered as viable lesions, while lesions in stadiums IV–V where considered as non-viable lesions as described by Eisele et al. [1].

**Table 1:** Fortaleza classification

Stages		Symptom	Parasite activity	Time period
Penetration	Stage I	Erythema	Penetration	3–7 h
Beginning hypertrophy	Stage II	Pruritus, pain, erythema around central dark dot	Beginning hypertrophy	Days 1–2 after penetration, lasting 2–3 days
White halo	Stage IIIa	White halo surrounding the black dot, faecal coils, egg expulsion	Hypertrophy into a sphere	Days 2–3 after penetration, lasting 3–4 days
	Stage IIIb	White halo, caldera formation, loss of firmness, faecal coils, egg expulsion	Development of a rim surrounding the rear cone	Days 6–7 after penetration, lasting 2 weeks
Involution	Stage IVa	No egg excretion, wrinkled lesion	Shrinking hypertrophy, dying parasite	3–4 weeks after penetration, lasting 1–2 weeks
	Stage IVb	Necrotic, dissected lesion	Dead parasite	4–6 weeks after penetration, lasting 1–2 weeks
Residuum	Stage V	Circular depression in the stratum corneum	No parasite	6–7 weeks after penetration for several months or permanent

Crater-like sores caused by manipulation with sharp instruments as well as suppurated lesions were documented. The localization of each lesion was noted on a visual scheme. Clinical pathology was assessed adapting the previously validated severity scores for acute and chronic tungiasis, but the assessment was limited to the index lesion [14]. This resulted in a modified severity score for acute tungiasis (mSSAT) and a modified severity score for chronic tungiasis (mSSCT). The mSSAT ranges from 0 to 7 and the mSSCT 0 to 6 points. Intensity of pain and pruritus were assessed using a visual analogue scale (VAS) varying from 0 to 4 points. Lesions were photographed with a digital camera equipped with a macro-objective (Canon EOS 450 D, Tokyo, Japan) and a macro-flash. The macro-objective had a magnification ratio of 1:1.

HRIT was performed with a FLIR T 660bx camera which uses a 320 × 240 pixels uncooled microbolometer focal plane array to detect infrared rays (FLIR Instruments, Wilsonville, OR, USA). The camera has the following optical image specifications: the lens has a field of view (FOV) of 25° × 19° with a minus focus distance of 0.4 m and a spatial resolution in field of view (IFOV) of 1.36 mRad. The camera also allows taking digital photos with a separate objective next to the infrared lens. However, the resolution of digital pictures is rather low; therefore, this tool was not used for study purposes.

Thermographic images were taken following a standard protocol developed by HF, MT and AS. When possible, the foot was placed on a neutral ground. The temperature and relative air humidity were noted. The camera was held at about 1 m from the foot, then the lesion of interest was focused and the thermographic picture was taken.

### Data analysis

The temperature matrix generated by the infrared camera was transformed using the program FLIR Quick Report (version 2.1 SP2, FLIR Instruments, 2009, Wilsonville, OR, USA). Before analysis, air temperature and relative humidity were entered. The program then adjusted the thermographic map with regard to temperature and humidity.

The program allows four visual transformations of the thermographic matrix. The so-called iron transformation was used as standard colour rendering. In a second step, the so-called rainbow transformation was used enabling the rendering of lower temperatures in blue-coloured spectrum. In a third step, an inverted grey transformation was generated facilitating the comparison of hot versus cool areas of the skin.

To assess changes of skin temperatures after extraction of embedded sand flea, we developed an algorithm to compensate for differences in skin radiation, which were not related to inflammation. In the thermal image, a standardized area around the lesion was selected using FLIR Quick Report. Temperature pixels of the selected area were transferred to an Excel spreadsheet (Microsoft Excel, Albuquerque, New Mexico 2010, USA), and the median of all pixels of the area was calculated. When several lesions on the same foot were present, an index area was selected. The isotherm area above the arithmetic mean was marked in green. The pre- and post-treatment isotherm areas above this cut-off were compared. The thermal image of the whole foot was processed in the same way.

Statistical analysis was carried out using SPSS for Windows (version 16, SPSS INC, Chicago, IL, USA). Since data were not distributed normally, we used median, interquartile range (IQR) and range to describe central tendency and data dispersion, respectively. Spearman rank correlation coefficient, the chi-squared test and the Wilcoxon signed-rank test were used when appropriate.

## Results

Thirty-six patients with tungiasis lesions on their feet were included in the study. The demographic, clinical and parasitological characteristics of the patients are shown in Table 2. Patient RNR was excluded from the thermographic analysis since both feet were severely inflamed, and the identification of a circumscriptive inflamed area around a defined lesion was not possible.

**Table 2:** Demographic, clinical and parasitological characteristics of the patients (*N* = 36) and lesions (*N* = 71)

Characteristics of patients (*N* = 36)	
Age in years, median (IQR)	14.5 (10.25–18.75)
Female/male	20/16
Number of lesions, median (IQR/min-max)^a^	1.5 (1.0–2.0/1–250)
mSSAT, median (IQR)^b^	2.0 (0.0–4.0)
mSSCT, median (IQR)^b^	1.0 (0.0–3.0)
Presence of pain^b^, *N* (%)	18/36 (50.0%)
Presence of pruritus^b^, *N* (%)	17/36 (70.8%)
Deformed/lost toe nails, *N* (%)	11/36 (16.9%)
Characteristics of lesions (*N* = 71)^a^	
Viable lesions (stages I–III)^b^, N (%)	59/71 (83.1%)
Non-viable lesions (stages IV–V)^b^, *N* (%)	5/71 (7.1%)
Manipulated lesions^b^, *N* (%)	7/71 (9.8.0%)
Lesions with acute local inflammation^c^, *N* (%)	59/71 (83.1%)
Lesions with chronic local inflammation^d^, *N* (%)	54/71 (76.1%)
Lesions with bacterial superinfection^e^, *N* (%)	13/71 (18.4%)
Presence of fissures, *N* (%)	8/71 (11.3%)
Presence of ulcers, *N* (%)	9/71 (12.7%)

^a^Patient RNR was excluded from analysis as the number of lesions was not countable

^b^See the ‘Methods’ section

^c^Lesion presenting with desquamation and/or oedema and/or erythema and/or warmness and/or ulcer and/or fissure

^d^Lesion presenting with hyperkeratosis and/or hypertrophic nail rim and/or nail deformation and/or lost nail and/or chronic oedema and/or scar

^e^Presence of pustules, suppuration or an abscess

The median temperature of the lesion measured by HRIT correlated significantly with the degree of acute and chronic inflammation (rho = 0.380, *p* = 0.022, and rho = 0.337, *p* = 0.044, respectively) and the intensity of localized pain (rho = 0.395, *p* = 0.017) but not with local pruritus (rho = 0.068, *p* = 0.69). The stage or viability of the lesion did not correlate significantly with the temperature of the lesion. The median temperature of the affected area was 29.5 °C (IQR 28.1–30.9 °C) before treatment, while the median temperature of the affected foot was 27.1 °C (IQR 25.1–27.9 °C) (rho = 0.480, *p* = 0.003). Four days after surgical extraction of the embedded sand flea, the temperature of the affected area decreased significantly to a median of 20.9 °C (IQR 17.8–23.7 °C) (rho = 0.457, *p* = 0.005). The clinical and thermographic characteristics of the patient are summarized in Table 3.

**Table 3:** Demographic, clinical and thermographic characterization of the patients (*N* = 36); patients described in detail are marked in italics

Initial patient	Sex	Age	Foot	Number of viable/nonviable/manipulated lesions	Stage of lesions	Topographic localization of lesions^a,b^	Signs of acute inflammation (mSSAT) in the index area^c^	Signs of chronic inflammation (mSSCT) of the index area^d^	Pain (0–4)	Temperature of the foot at day 0 (°C)^e^	Perilesional temperature at day 0 (°C) of the index area^e^	Perilesional temperature at day 4 (°C) of the index area^e^
BMR	F	14	L	1/0/0	1	10	0.5	0.5	2	29.34	31.62	–
			R	1/0/0	1	2	0.5	0.5	2	30.40	31.41	–
RMR	M	24	R	0/0/1	0	1	4.0	4.0.	2	27.46	28.38	–
OR	M	16	L	1/0/0	3b	1	3.0	3.0	1	25.36	29.19	–
AMR	F	17	R	1/0/0	3a	8	3.0	1.0	3	29.71	31.77	–
BLS	M	13	R	1/0/0	3b	2	2.0	3.0	2	21.42	22.31	–
CER	M	6	L	0/0/3	0	8/8/9	3.0	2.0	4	21.35	24.59	–
MMR	F	20	L	1/0/0	3a	10	2.5	0.0	3	24.01	24.34	–
RJR	M	15	L	1/0/0	2	11	1.0	1.0	0	22.47	24.83	–
DSS	M	10	L	2/0/0	2,3a	7/8	2.0	1.0	2	26.23	27.26	–
SR	F	13	R	1/0/0	3a	8	4.0	2.0.	3	26.52	29.58	–
RDS	M	27	L	1/0/0	2	5	6.0	5.0	3	27.98	29.27	–
			R	1/0/0	3a	2	2.0	5.0	3	28.42	32.98	–
ENR	M	18	L	1/0/0	3a	6	0.0	2.0	0	27.09	27.79	–
AGR	F	12	L	0/1/0	4a	1	1.5	1.0	0	27.06	29.19	–
ELR	F	8	R	1/1/0	2/4a	9/10	0.0	1.0	0	22.98	23.96	–
*CMR*	*F*	*19*	*R*	*0/0/2*	*0*	*1/6*	*0.0*	*4.0*	*0*	*27.49*	*29.33*	–
*TAR*	*F*	*21*	*L*	*1/0/0*	*3a*	*2*	*0.0*	*5.0*	*0*	*27.67*	*28.77*	–
			*R*	*2/0/0*	*2*	*3*	*0.0*	*3.0*	*0*	*27.43*	*29.46*	–
BDR	F	23	L	0/2/0	4b	1	0.0	5.0	0	26.82	27.87	–
MGR	F	5	L	2/0/0	1,3a	4	0.0	0.0	0	22.33	22.26	–
TMR	F	17	L	1/0/0	2	11	2.0	0.0	3	21.66	21.84	–
DRJ	F	16	L	2/0/0	3a,3b	3/11	1.0	0.0	0	27.18	27.29	–
MNR	M	15	L	1/0/0	2	10	0.0	0.0	0	28.18	28.52	–
JNR	F	20	L	1/0/0	3a	11	0.0	0.0	0	23.33	24.05	–
*RNR*	*M*	*55*	*L/R*	*-*		*Whole foot*	*7.0*	*6.0*	*4*	**-**	**-**	–
BDR	M	16	L	1/0/0	2	8	0.0	0.0	0	27.66	28.77	–
AAR	F	11	L	1/0/0	3a	9	0.0	0.0	0	25.70	28.30	–
			R	1/00	3a	10	0.0	0.0	0	24.78	26.44	–
*NMS*	*M*	*23*	*L*	*2/0/0*	*2*	*1*	*2.0*	*4.02.5*	*2*	*27.79*	*30.52*	–
			R	3/0/0	3a	5	6.0	4.0	3	*27.41*	*30.45*	–
*JMR*	*M*	*10*	*L*	*4/0/1*	*2,3a*	*3/4/8/* *5*	*4.0*	*5.0*	*2*	*32.57*	*33.42*	
*YAR*	*F*	*12*	*L*	*4/0/0*	*3b,2*	*1* */2*	*3.0*	*3.0*	*1*	*30.29*	*31.03*	*17.10*
*MRR*	*F*	*13*	*L*	*6/1/0*	*2,3a,3b/4b*	*6/7* */11*	*4.0*	*2.0.*	*4*	*25.90*	*28.10*	*24.30*
ASB	F	7	L	1/0/0	3b	6	4.0	1.0	2	28.26	30.67	20.91
ELR	F	5	R	2/0/0	3a,3b	10/11	4.0	1.0	1	25.82	28.03	20.96
EBR	M	6	R	6/0/0	2,3a,3b	6/7/12	4.0	2.0	1	27.93	30.87	28.42
AMR	F	12	L	1/0/0	1	3	0.0	0.0	0	28.34	29.06	23.34
JNR	M	7	L	1/0/0	1	3	2.0	1.0	0	29.41	30.00	18.10
EMR	F	16	L	1/0/0	2	10	2.0	1.0	0	26.65	28.45	23.50
DR	F	12	L	1/0/0	1	10	1.0	1.0	0	25.25	27.55	20.70

^a^If more than one area was affected, an index area was selected for thermographic evaluation (index area underlined)

^b^(1) toe nail rim = 1, (2) toe nail rim = 2, (3) toe nail rim = 3, (4) toe nail rim = 4, (5) toe nail rim = 5, (1) toe sole = 6, (2) toe sole = 7, (3) toe sole = 8, (4) toe sole = 9, (5) toe sole = 10, foot sole = 11, heel = 12, medial foot rim = 13, lateral foot rim = 15

^c^Taking into consideration the index lesion (presence of oedema, erythema or warmness = 1 point, ulcer = 1 point, fissure = 1 point, pustule/abscess = 1 point, suppuration = 1 point, pruritus = 0.5 point, pain = 0.5 point, severe pruritus (≥3 VAS) = 1 point, severe pain (≥3 VAS) = 1 point)

^d^Taking into consideration the index lesion (hyperkeratosis = 1 point, chronic oedema = 1 point, hypertrophic nail rim = 1 point, deformed toe nail = 1 point, loss of nail = 1 point, scar/deformed toe = 1 point)

^e^See the ‘Methods’ section

Patients with similar clinical characteristics were grouped, and seven patients representing the entire spectrum of clinical pathology were selected for detailed description. Clinical and thermographic characteristics of these patients are detailed below.

### Patient CMR

#### Left foot

**Fig. 1:**
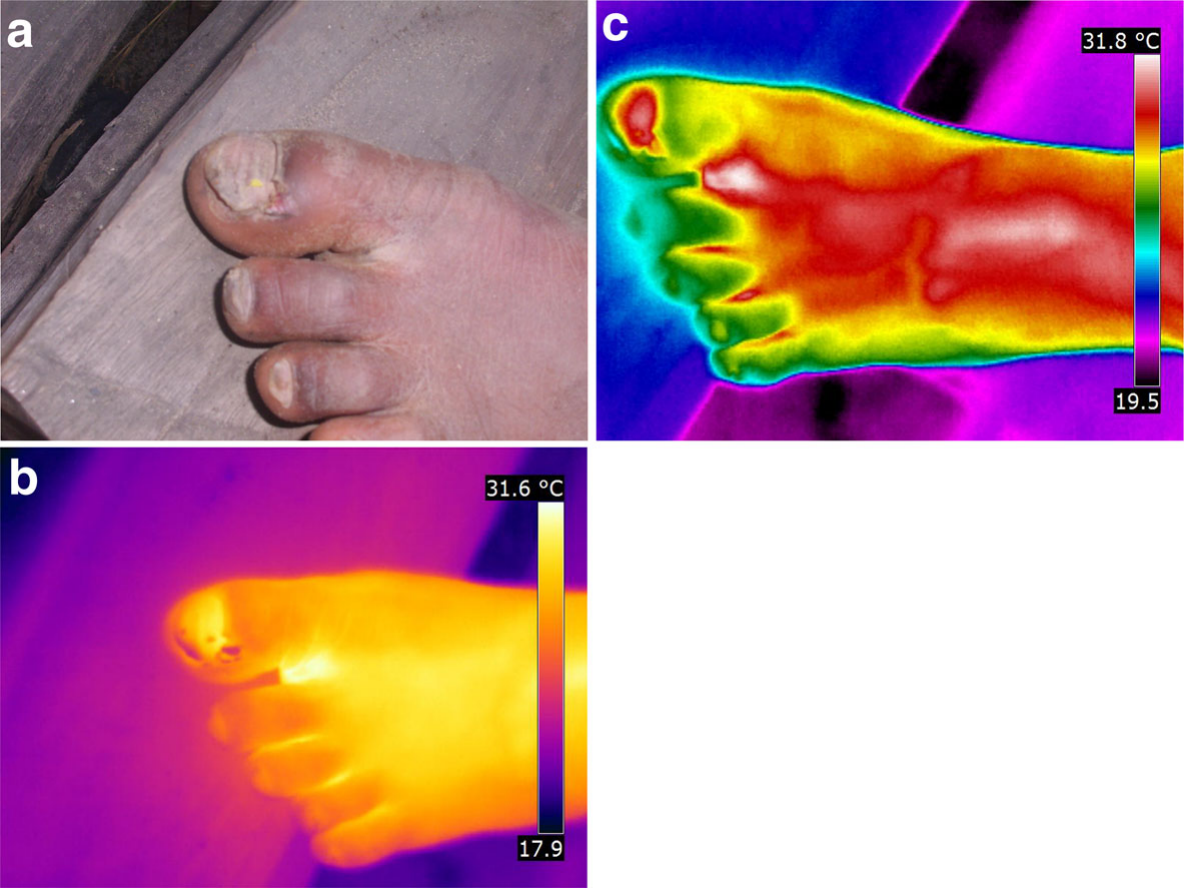
(**a**) left foot with two manipulated lesions at the medial nail fold of the first toe and at the base of the first and second toe. (**b**) left foot, hyperthermic area s at the medial nail fold of the first toe and the base of the first and second toe (iron transformation). (**c**) left foot, hyperthermic area s at the medial nail fold of the first toe and the base of the first and second toe (rainbow transformation)

The patient [Fig. 1a] has two lesions on the left foot. One manipulated lesion is located at the lateral distal edge of the nail of the first toe, where a triangular sore had developed. The toe presents signs of chronic inflammation (hyperkeratosis, hypertrophic nail rim and nail deformation). The yellow area on the nail of the first toe is a rest of nail polish. A second manipulated lesion is hidden in the interdigital space between the first and the second toe.

The iron transformation of the thermal image shows two hyperthermic areas: one around the lesion in the interdigital space and another below the proximal part of the nail and along the proximal nail fold of the first toe [Fig. 1b]. The lesion in the interdigital space was much more evident by thermography then by clinical assessment.

The inflamed areas were even more obvious in the rainbow transformation [Fig. 1c]. The inflammation visualized by the thermographic image clearly exceeded the clinically visible inflammation. The hypothermic area in the centre of the sore corresponds to the residual nail polish [Fig. 1b].

### Patient TAR

#### Left foot

**Fig. 2:**
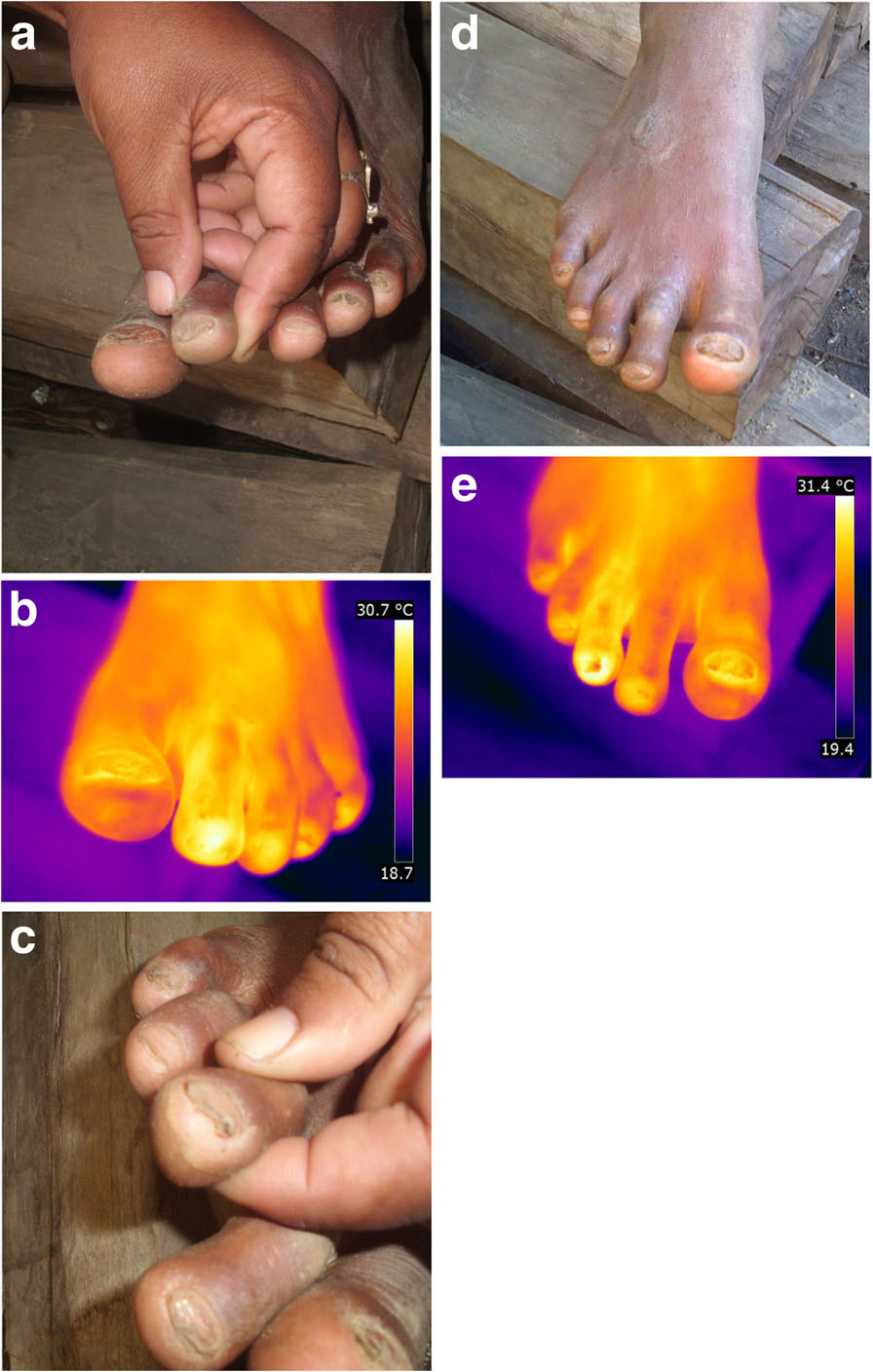
(**a**) left foot with stage IIIa lesion and with periungual edema. (**b**) left foot, hyperthermic areas on all toes (iron transformation). (**c**) right foot whit 2 stage II lesions with suppurated and uplifted nail. (**d**) right foot overview with hypertrophic nails and oedematous nail rims. (**e**) right foot hyperthermic areas on the first and third toe (iron transformation)

On the left foot, all toes present signs of previously penetrated sand fleas. The shape of the nail of the first toe indicates repeated infections. The nail of the second toe is also distorted. One sand flea in stage IIIa had penetrated at the distal lateral nail rim [Fig. 2a]. The faecal coil expelled by the flea indicates that the parasite was metabolically active. Oedema and brilliant skin reflect the chronic nature of the inflammation. The third toe shows a slight periungual oedema. The nail of the fourth toe is deformed and surrounded by periungual oedema; the desquamated skin indicates a chronic inflammation. The nail of the fifth toe is slightly deformed.

The iron transformation shows hyperthermic areas on all toes [Fig. 2b]. There is an impressive hyperthermic area at the second toe extending towards the metatarsophalangeal articulation. The denuded nail bed of the first toe shows small hypothermic areas surrounded by hyperthermic areas.

#### Right foot

Two viable lesions in stage II are located on the third toe on the medial and lateral side of the distal nail rim. The lesions are erythematous and hyperthermic; an extended area around the lesions is desquamated. The nail itself is deformed and uplifted and shows fissures and suppuration [Fig. 2c]. The nail of the first toe is deformed, and the nail rim is hypertrophic. The surrounding skin is oedematous and shiny [Fig. 2d].

The iron transformation of the thermal image shows hyperthermic areas on the first and third toe. The inflammation is particularly prominent at the third toe: it extends towards the metatarsophalangeal articulation and continues until the metatarsus. The hypothermic area at the tip of the third toe corresponds to the uplifted nail. The first toe presents with hypothermic islets on the denuded areas of the nail bed [Fig. 2e].

### Patient RNR

#### Left foot

**Fig. 3:**
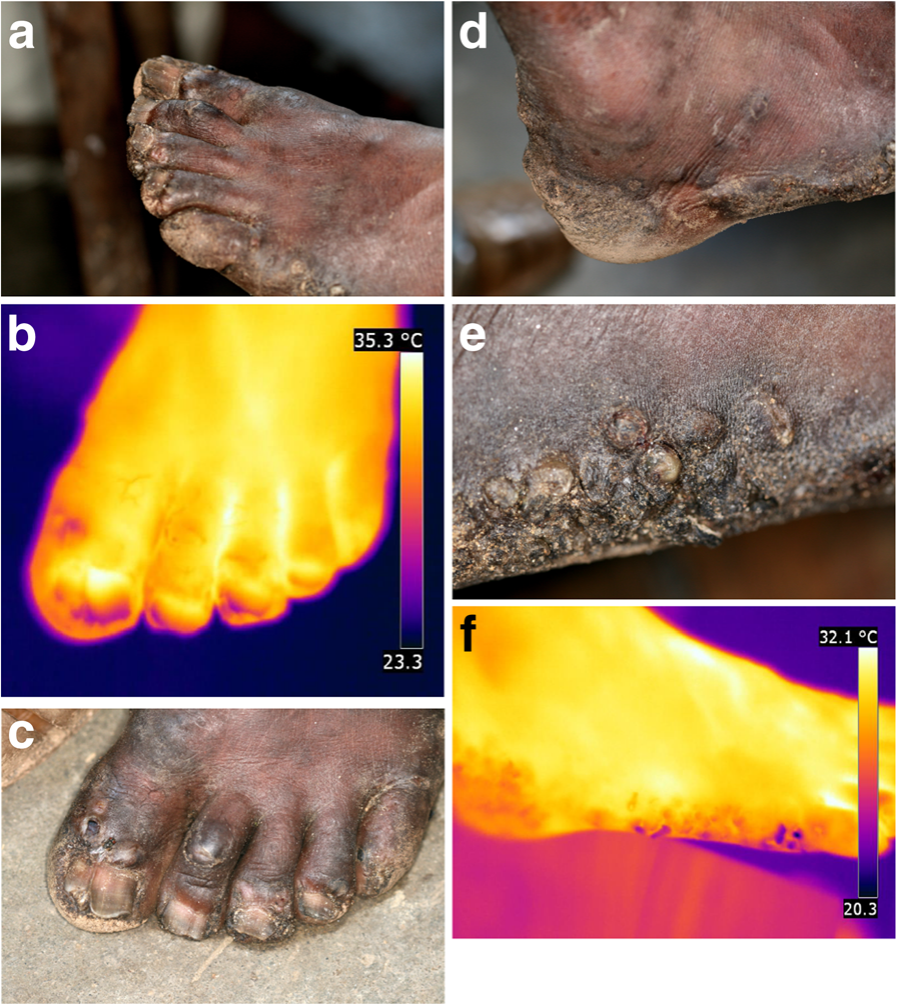
(**a**) left foot with hyperkeratosis und desquamation presenting lesion in all stages. (**b**) left foot, hyperthermia of the whole foot except for the areas corresponding to uplifted nails (iron transformation). (**c**) left foot detail, fly feeding on a lesion of the first toe. (**d**) right foot, viable lesions hidden under hyperkeratotic skin. (**e**) right foot, extensive inflammation and hyperkeratosis of the heel and lateral rim. (**f**) right foot, hypothermic areas on the lateral rim corresponding to hyperkeratosis ( iron transformation)

The patient suffered from repeated sand flea infections for several months. To avoid new infections, the patient wore shoes day and night, and therefore, some of the lesions were crusted to the shoe. While removing the shoe, the crusts disrupted and created painful sores. All toes of both feet are affected; nails are deformed or lost and nail rims are distorted. There is considerable hyperkeratosis and desquamation; scars are visible on all toes; fissures and ulcers are present; most lesions are superinfected and present suppuration. Lesions of all stages are present. Partially, the skin is hyperpigmented [Fig. 3a].

The iron transformation shows that the whole foot, except the edges of the distorted nails, are extremely hyperthermic [Fig. 3b]. Relatively cold areas can be seen at the nail rims and on the medial side of the first toe where a fly was feeding on a lesion [Fig. 3b, c].

#### Right foot

At the right foot, acute and chronic lesions overlap. Parts of the skin are hyperkeratotic. In several lesions of the foot, viable tungiasis lesions are hidden under the hyperkeratotic skin [Fig. 3d]. Areas of hyperpigmentation alternate with areas of hypopigmentation. The extensive inflammation of the heel and the lateral rim of the foot is clearly visible [Fig. 3e]. In the iron transformation, distinct hypothermic areas at the lateral rim are visible [Fig. 3f]. They correspond to hyperkeratotic areas resulting from multiple previous infections.

The patient presents more than 200 lesions on both feet.

### Patient NMS

#### Left foot

**Fig. 4:**
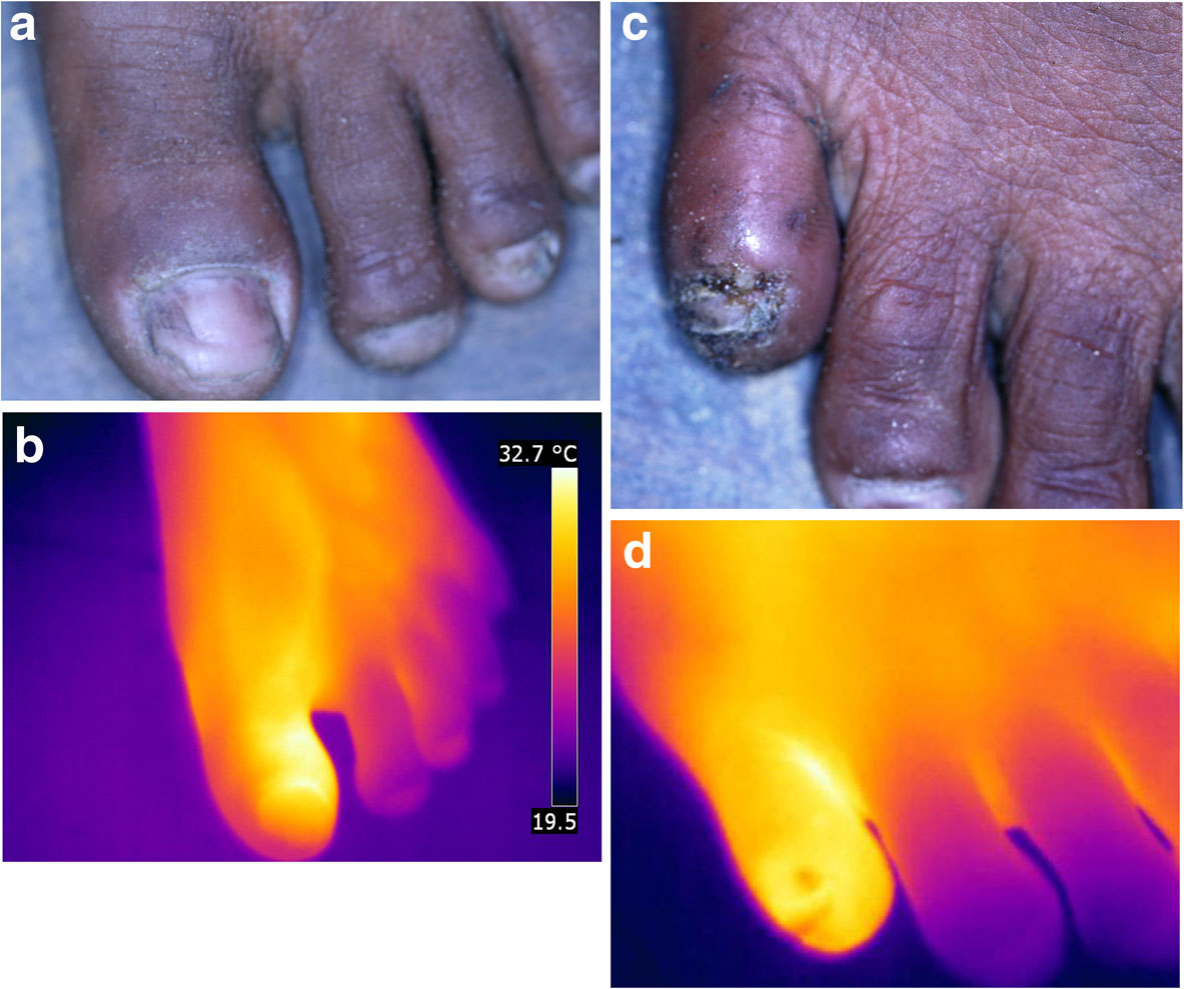
(**a**) left foot, two stage II lesions. (**b**) left foot, hyperthermia form the tip of the toe till the proximal interphalangeal articulation (iron transformation). (**c**) right foot, three stage IIIa lesion on the medial nail rim of the fifth toe. (**d**) right foot, hyperthermia form the tip of the toe till the metatarsus (iron transformation)

The patient has two embedded sand fleas at the lateral proximal part of the nail rim of the first toe. The lesions are in stage II [Fig. 4a]. Only a slight erythema is visible; warmness was palpable. The thermal image shows a considerable hyperthermia of the first toe extending from the tip of the toe to the proximal interphalangeal articulation [Fig. 4b].

#### Right foot

On the right foot, the patient has three embedded sand flea lesions on the nail rim of the fifth toe (stage IIIa). The lesions are hyperthermic and oedematous; the nail rim is inflamed; a small ulcer, a fissure and suppuration are present. The nail is deformed and partially uplifted [Fig. 4c].

In the thermal image, the inflammation extends from the tip of the toe to the metatarsus. A tiny hypothermic area is visible at the edge of the nail rim where the nail is deformed and uplifted [Fig. 4d].

### Patient JMR

#### Left foot

**Fig. 5:**
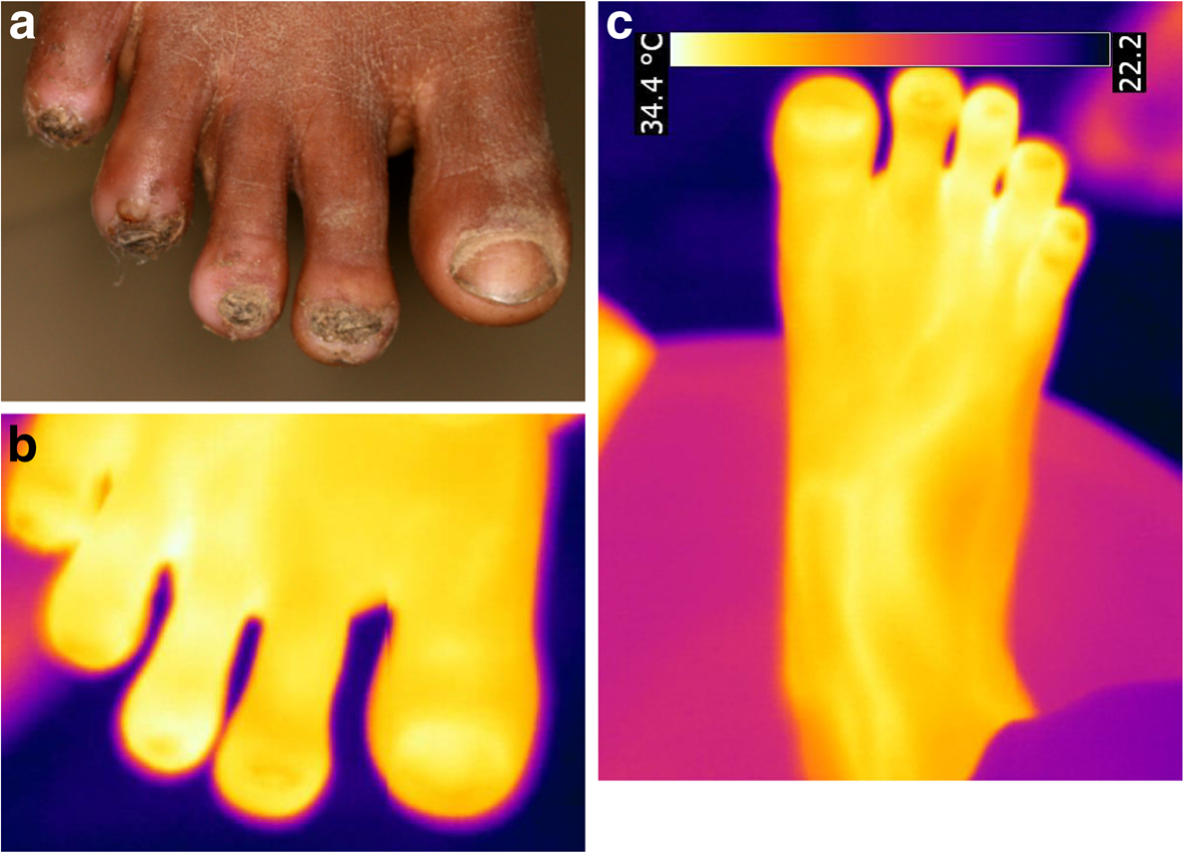
(**a**) left foot, multiple stage II and III lesions on the fourth and fifth toe. (**b**) left foot, extreme hyperthermia of the third and fourth toe (iron transformation). (**c**) left foot, hyperthermia extending form the distal end of the toe till the metatarsus (iron transformation)

The patient had several deformed or lost nails due to repeated tungiasis. Partially, the periungual skin is desquamated; erythema and oedema are present. The remaining proximal and lateral nail folds are hypertrophic. Hyperkeratosis and skin excoriation indicate repeated manipulation with a sharp instrument. An abscess had formed around an embedded sand flea at the nail wall of the medial third toe; the lesion shows signs of manipulation. The fourth toe presents a stage IIIa lesion in the proximal edge of the formal nail plate and two stage II lesions in both proximal edges of the nail plate. The fifth toe presents a stage II lesion on the medial nail wall [Fig. 5a].

The thermographic image shows that the third, fourth and fifth toe are extremely hyperthermic [Fig. 5b]. The inflammation extends from the distal end of the toes to the metatarsus [Fig. 5c].

### Patient YAR

#### Left foot before treatment

**Fig. 6:**
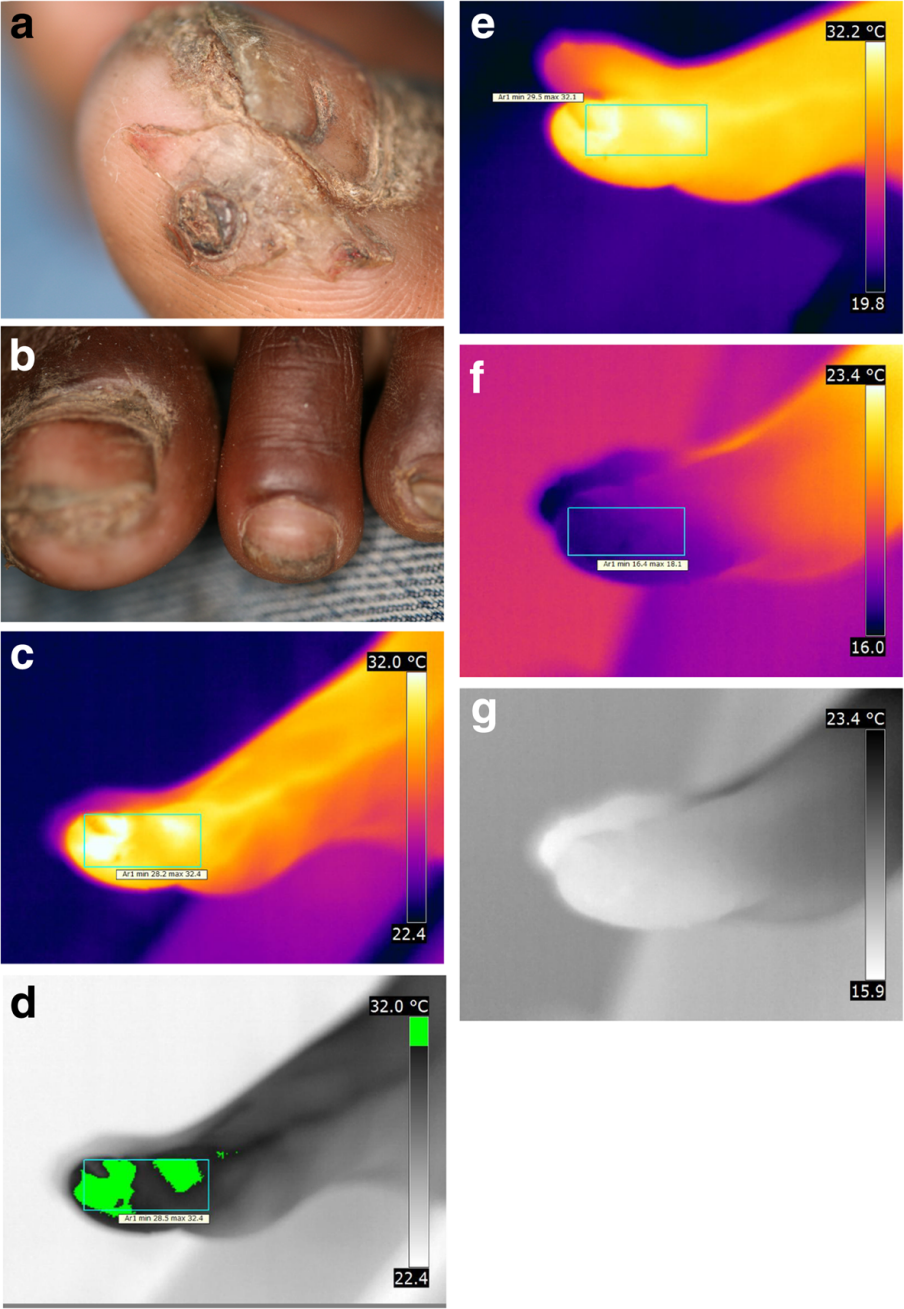
(**a**) left foot, multiple stage II and IIIb lesions together with hyperpigmentation and hyperkeratosis. (**b**) left foot, stage II lesion on the proximal medial edge of the second toe. (**c**) left foot, perilesional hyperthermia with a median of 31.0 °C (iron transformation). (**d**) left foot, perilesional hyperthermia with green transformation of isotherm areas ≥ 31.0 °C (inverted grey transformation). (**e**) left foot, perilesional hyperthermia immediately after extraction, median of 31.2°C (iron transformation). (**f**) left foot, perilesional hypothermia four days after extraction, median 17.1°C (iron transformation). (**g**) left foot perilesional hypothermia in inverted grey (inverted grey transformation)

The patient has three sand flea lesions at the medial rim of the nail of the first toe. A stage IIIb lesion appears desquamated without inflammatory signs, but with some uplifted hyperkeratotic layers of surrounding epidermis. Some millimetres proximal of the IIIb lesion, two lesions in stage II are present. The distal edge of the nail plate presents with hyperkeratosis and hyperpigmentation testifying the presence of previous tungiasis infections [Fig. 6a]. Another stage II lesion is located at the proximal medial edge of the nail plate of the second toe [Fig. 6b]. The first and second toes are extremely hyperthermic with an arithmetic median of 31.0 °C (min-max 28.5–32.4 °C). This results in perilesional coloration in green of isotherm areas above 31.0 °C in the inverted grey image [Fig. 6c, d].

#### Left foot after treatment

Immediately after surgical extraction of the sand fleas, the temperature of the perilesional area raised to 31.2 °C (min-max 29.2–32.1 °C) [Fig. 6e]. Four days after extraction, the iron transformation shows a drastic reduction of the temperature in the affected area to a median of 17.1 °C (min-max 16.4–18.1 °C) [Fig. 6f]. In the inverted grey image, green areas, indicating an isotherm above 31.0 °C, could not be identified [Fig. 6g].

### Patient MRR

#### Left foot before treatment

**Fig. 7:**
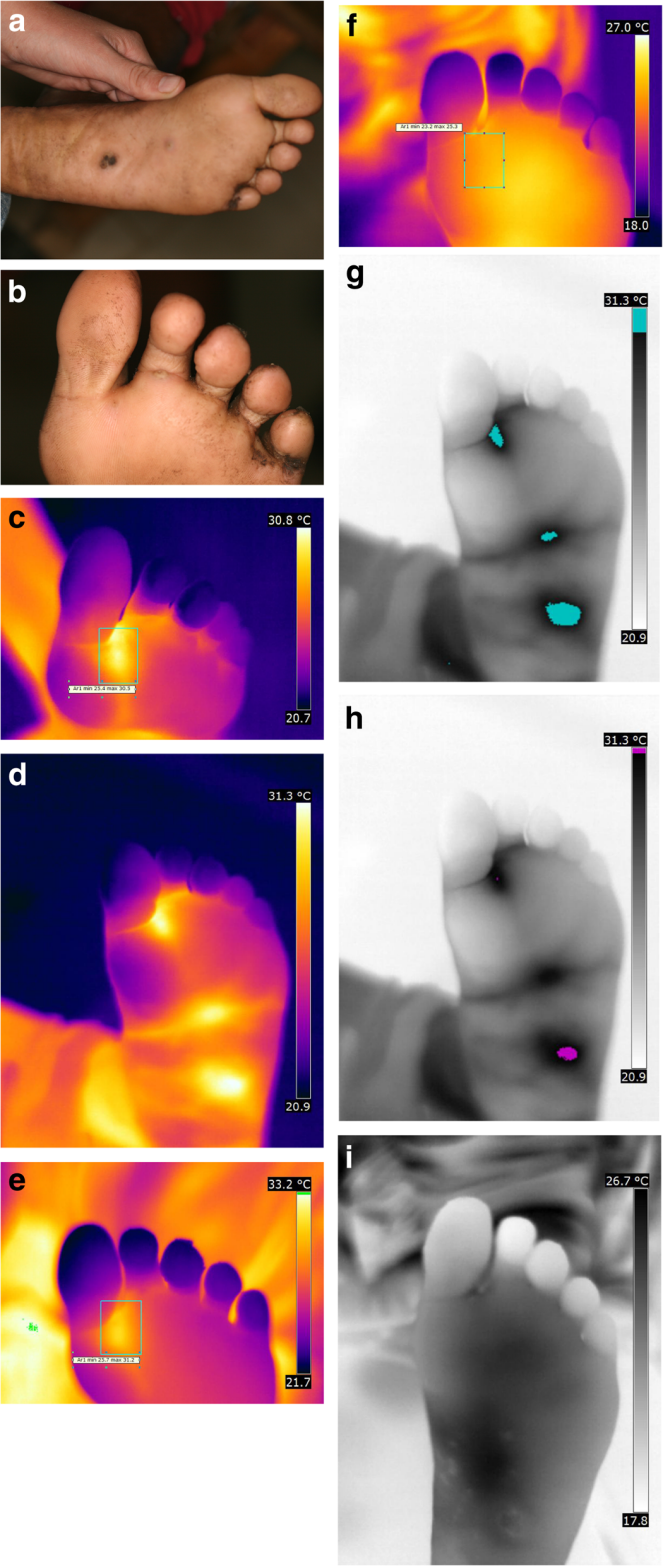
(**a**) left foot, lesions stage IIIb and IVb at the mid tarsal joint and one lesion stage IIIa at the center of the sole. (**b**) left foot, two viable lesions at the base between the first and second toe. (**c**) left foot, rhomboid hyperthermic area at base between the first and second toe, median temperature 28.1°C( iron transformation). (**d**) left foot, hyperthermic areas around the lesions (iron transformation). (**e**) left foot, hyperthermic area at the base between the first and second toe immediately after extraction, median temperature 29.2°C (iron transformation). (**f**) left foot, hyperthermic area at the base between the first and the second toe immediately after extraction four days after extraction, median temperature 24.3 °C (iron transformation). (**g**) left foot, overview with isotherm areas > 30.5 °C marked in green (inverted grey transformation). (**h**) left foot, overview with isotherm areas >31.2 °C marked in green (inverted grey transformation). (**i**) left foot four day after extraction, isotherm areas >30.5°C are no longer present (inverted grey transformation)

The patient presents seven lesions at the sole of the left foot [Fig. 7a, b]. A stage IIIa lesion is located in the centre of the sole. The embedded sand flea is surrounded by a distinct erythema. At the level of the mid-tarsal joint, a cluster of three sand fleas is visible. They are in stages IIIb and IVb and covered by a brownish crust [Fig. 7a]. A stage II lesion is localized between the first and the second toe; a stage IIIa lesion is present at the base of the second toe. Both lesions go along with oedema, erythema and hyperthermia; the lesion on the second toe appears shiny and stretched. Another stage II lesion is present between the forth and the fifth toe without showing inflammatory signs. Hyperkeratosis and scars representing residues of previous sand flea infestations can be seen at the base of the fifth toe. The dark material around the base of the fifth toe corresponds to faecal traces from the parasite dispersed in skin papillae [Fig. 7b]. The thermal imaging shows a rhomboid hyperthermic area at the base of the first and second toe extending to the metatarsophalangeal articulation [Fig. 7c]. When comparing the photograph with the thermal image, the hyperthermic area around the stage II lesion between the first and second toe is considerably larger than the visible erythema [Fig. 7a, d]. The hyperthermic area at the level of the mid-tarsal joint corresponds to the cluster of sand fleas [Fig. 7d].

#### Left foot after treatment

Immediately after extraction of the parasites at the base of the second toe, the temperature of the area raised from an arithmetic median of 28.1 °C (min-max 24.5–30.5 °C) to 29.2 °C (min-max 25.7–31.2 °C) at the site of manipulation [Fig. 7c, e]. Four days after extraction, the mean temperature of the previously affected area decreases to 24.3 °C (min-max 23.2–25.5 °C) [Fig. 7f]. In the overview inverted grey image, all perilesional areas have an isotherm temperature above 30.5 °C [Fig. 7g] but only in the cluster of lesions at the mid-tarsal joint the isotherm temperature reaches 31.2 °C [Fig. 7h], suggesting a higher perilesional temperature when lesions present in clusters. Four days after treatment, there were no more areas with isotherms above 30.5 °C [Fig. 7i].

## Discussion and conclusions

Since long, it is known that inflammation of the skin and of deeper tissue layers is characterized by pain, erythema, oedema and hyperthermia. Hyperthermia can be visualized as hot spots or as asymmetrical patterns in infrared thermography [15]. The software of the IR camera converts radiance measurements into temperatures by using the known emissivity of a target object and applying internal calibration. In 1998, Jones recognized the potential use of infrared thermal imaging with regard to skin diseases [16]. But only through the transfer of military technology, HRIT was introduced as a new tool for diagnosis and morbidity assessment, first in race horses and later in humans [17, 18]. Since then, HRIT has been used for diverse medical purposes, e.g. in detection of breast cancer, rheumatic disease or peripheral neuropathy in diabetic patients [19].

Hitherto, HRIT has been rarely used in inflammatory skin diseases of infectious origin. Ammer et al. applied whole body HRIT to measure asymmetry of skin temperature in patients with acute herpes zoster and post-herpetic neuralgia [20]. Arenas et al. used long-distance infrared thermal imaging for the diagnosis of disseminated mange (animal scabies) in wild Pyrenean goats [12].

By evaluating HRIT with a handheld thermography camera for the assessment of inflammation in patients with tungiasis, we follow the recommendation of the World Health Organization to develop innovative and intensified methodologies for the management of neglected tropical diseases (http://apps.who.int/iris/bitstream/10665/69660/1/WHO_CDS_NTD_IDM_2007.2_eng.pdf?ua=1).

We showed that inflammation is displayed by an increased surface temperature of the affected area. Thereby, HRIT offers the possibility to assess inflammation in a quantitative and objective manner, a progress compared to current methods to detect inflammation in parasitic skin diseases [14, 21–23]. Our findings corroborate previous observations showing that the area of inflamed skin due to a bacterial infection is much more precisely determined by HRIT than by visual inspection alone [24]. Interestingly, we found a significant association of perilesional skin temperature not only with the score assessing signs of acute inflammation but also with the score assessing chronic inflammation. This could indicate a role for HRIT in assessing chronic skin disease such as cutaneous leishmaniasis, a disease for which reproducible methods to measure the outcome of therapeutic interventions is urgently needed [25].

Moreover, accuracy of thermal imaging was proven when lesions were hidden and missed by the clinical examination. Although we did not analyse it systematically, our findings suggest that perilesional temperatures were higher when lesions occurred in clusters as compared to isolated lesions.

Early stage lesions (stages I and II of the Fortaleza classification) which are generally more difficult to detect because of limited morphological changes were easily identified by thermal imaging. This corroborates the previous findings wherein thermal imaging was used to detect early inflammation. In horses, HRIT could help identifying spot flexor tendon injuries before there were any visible signs of lameness [17]. Similarly, in patients with recurrent herpes labialis, a marked increase in temperature was observed prior to the formation of vesicles [26].

A common symptom in tungiasis is a boring pain at the site of the lesion which develops early after penetration [1]. If the pain affects particularly sensitive areas, such as the nail wall or the heel of the foot, it is incapacitating and keeps children from attending school and adults from going to work or to perform their household chores [4]. Our data show that localized intensity of pain is reflected by high temperature of the lesion. These findings are similar to observations in herpes zoster where the thermal patterns correlated significantly with the intensity of pain [27]. HRIT provided a good discrimination between patients with scleroderma spectrum disorders and those with primary Raynaud’s phenomenon, both autoimmune diseases which present different pain characteristics [28].

Thermal imaging before and after adequate surgical extraction of embedded sand fleas showed a temperature increase of the perilesional area immediately after the extraction of the embedded parasite [Figs. 6c, e and Figs. 7c, e, g and h] and then a perilesional temperature decrease [Figs. 6f, h and Figs. 7f, i]. It is plausible that the surgical extraction per se causes immediate inflammation, which is reflected by a higher temperature in HRIT. When inflammation had resolved after a couple of days, perilesional skin temperature was low compared to the median temperature of the intact foot. Our hypothesis is that after regression of inflammation, microcirculation in the skin is downregulated causing, thereby, a slight hypothermia. Interestingly, serial thermal imaging in a mild case of herpes zoster at the thorax showed a correlation between decreasing temperature, decreasing pain and macroscopic healing of the lesions [27]. In a patient with leprosy, Vargas et al. proved that HRIT is more sensitive than clinical examination in monitoring the regression of hypothermic skin areas during treatment [29].

Taken together, we think that HRIT is a useful tool to study tungiasis-associated inflammation and to monitor regression of clinical pathology after treatment. Additionally, HRIT may be of value for the detection of early or atypical lesions that are difficult to detect by the naked eye.

Our findings suggest that high-resolution HRIT can be applied to inflammatory skin diseases, irrespective whether the infectious agents are ectoparasites, protozoa, helminths, bacteria, viruses or fungi. Tropical diseases in which thermal imaging may provide useful information with regard to early detection, monitoring or disease progression and/or treatment are listed in Table 4. While most mentioned skin diseases probably go along with hyperthermia, in leprosy and buruli ulcer, a decrease in temperature is to be expected.

**Table 4:** Tropical diseases for which HRIT could be used as a tool to measure morbidity and to monitor treatment success

Ectoparasites	Hookworm-related cutaneous larva migrans	Tungiasis	Scabies	Myiasis	
Helminths	Strongyloidiasis stercoralis	Onchocerciasis	Cistocercosis	Dracunculiasis	
Protozoa	Cutaneous leishmaniasis				
(Myco)bacteria	Leprosy	Cutaneous tuberculosis	Buruli ulcer	Noma	Bejel, yaws
Fungi	Tinea corporis	Tinea capitis	Mycetoma	Mycids	
Viruses	Herpes simplex	Herpes zoster	Papilloma virus		

Only recently, thermography cameras have become available at affordable prices. Therefore, handhold portable IR cameras or smartphones equipped with IR objectives could be used for thermal imaging in resource-poor settings even at the population level. Possibly, this method will facilitate measurements of disease burden for NTDs where this has been difficult so far.

Obviously, the study had several limitations. First, it is important to acknowledge that an isothermal temperature scale rendering will not be accurate unless all of the highlighted areas have the same emissivity and the ambient temperature is the same for all artefacts within the area. Second, the interpretation of thermographic images is difficult in patients with very extended and multiple lesions. Third, the interpretation of thermographic images is rather subjective [18]. Forth, since the study was conducted in a resource-poor rural community in Madagascar, it was not possible to standardize thermal measurements as it could have been done in a hospital setting in an industrialized country. This impairs the quantitative comparison of the degree of inflammation between different patients before and after treatment.
